# Factors affecting spatiotemporal patterns of nest site selection and abundance in diamondback terrapins

**DOI:** 10.1002/ece3.9866

**Published:** 2023-03-15

**Authors:** Patricia Levasseur, Robert Prescott, Mark Faherty, Chris Sutherland

**Affiliations:** ^1^ Department of Environmental Conservation University of Massachusetts‐Amherst Amherst Massachusetts USA; ^2^ Mass Audubon Wellfleet Bay Wildlife Sanctuary Wellfleet Massachusetts USA; ^3^ Centre for Research into Ecological and Environmental Modelling University of St Andrews St Andrews UK

**Keywords:** diamondback terrapin, nest selection, relative abundance, scale selection, spatiotemporal variation, visual headcount

## Abstract

Determining what factors influence the distribution and abundance of wildlife populations is crucial for implementing effective conservation and management actions. Yet, for species with dynamic seasonal, sex‐, and age‐specific spatial ecology, like the diamondback terrapin (*Malaclemys terrapin*; DBT), doing so can be challenging. Moreover, environmental factors that influence the distribution and abundance of DBT in their northernmost range have not been quantitatively characterized. We investigated proximity to nesting habitat as one potential driver of spatiotemporal variation in abundance in a three‐step analytical approach. First, we used a scale selection resource selection function (RSF) approach based on landcover data from the National Landcover Database (NLCD) to identify the scale at which DBT are selecting for (or avoiding) landcover types to nest. Next, we used RSF to predict areas of suitable nesting habitat and created an index of nest suitability (NSI). Finally, analyzing visual count data using a generalized linear mixed model (GLMM), we investigate spatiotemporal drivers of relative abundance, with a specific focus on whether similar factors affect offshore abundance and onshore nest site selection. We found the scale of selection for developed and saltmarsh land use classes to be 550 and 600 m and open water land use classes to be 100. Selection was positive for nesting areas proximal to saltmarsh habitat and negative for developed and open water. Expected relative abundance was best explained by the interaction between NSI and day of season, where expected relative abundance was greater within high NSI areas during the nesting season (2.20 individuals, CI: 1.19–3.93) compared to areas of low NSI (1.84 individuals, CI: 1.10–3.10). Our results provide evidence that inferred spatial patterns of suitable nesting habitats explain spatiotemporal patterns of terrapin movement and abundance.

## Graphical abstract

We investigated proximity to nesting habitat as one potential driver of spatiotemporal variation in abundance using nest location data and National Landcover Database landcover data in a resource selection function to predict suitable nesting habitat. Next, we used visual point count data and generalized linear mixed models to investigate spatiotemporal drivers of relative abundance, with a specific focus on whether similar factors affect offshore abundance and onshore nest site selection. We found selection for saltmarsh and beach habitats and avoidance of developed, open water and coniferous habitats for upland nest site selection and increased expected relative abundance adjacent to areas of highly suitable nesting habitat during the nesting season.

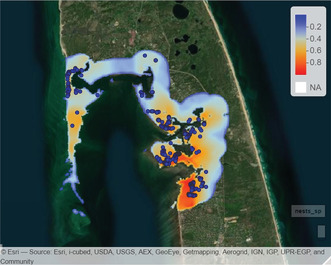

## 1 INTRODUCTION

Understanding where individuals within a population are distributed in space and time is a fundamental component of population ecology (Vandermeer & Goldberg, 2013) and studied in a wide range of taxa including fish (Nunn et al., 2014), birds (Fink et al., 2020), and mammals (Ehlers Smith et al., 2019). When the *where* is coupled with *when* and *how many*, we develop knowledge of spatiotemporal abundance patterns that can be applied to conservation and management strategies; particularly for rare and elusive species. Diamondback terrapins (DBT; *Malaclemys terrapin*) are listed as a vulnerable species on the International Union for Conservation of Nature (IUCN) red list, and in the United States, are protected or regulated in every range state (Kennedy, 2018; Roosenburg et al., 2019). However, despite their high conservation importance, there has been relatively limited progress in understanding what factors determine spatiotemporal distribution and abundance of this species. DBT are highly mobile and exhibit regular seasonal movement patterns and utilization of multiple habitats that vary by sex and age. For example, nearshore aquatic habitats are used by adults for winter brumation, spring breeding aggregations, and active season foraging (Butler et al., 2018; Castro‐Santos et al., 2019; Tucker et al., 2018). Females nest in uplands and hatchlings and juveniles use both upland and irregularly flooded high marsh habitats (Baker et al., 2018; Brennessel, 2006; Duncan & Burke, 2016). The survival, distribution, and abundance of DBT populations are impacted not only by direct threats associated with road, boat, and crab pot mortality (Chambers & Maerz, 2018; Crawford et al., 2014; Lester et al., 2018) but also by indirect threats to aquatic and terrestrial habitats as a result of development and climate change (Hartig et al., 2002; Roosenburg, 2018).

Areas of suitable, accessible nest habitat are required for successful recruitment and population persistence of nesting species. Female DBT prefer nest sites that are above the high tide line in loose sandy soils of coarse grain size, critical for proper gas exchange between the eggs and environment and successful embryonic development (Butler et al., 2018; Roosenburg & Place, 1995). Limited ground and overhead vegetation cover is also preferred, which not only maximizes solar exposure and reduces plant root egg mortality but also can increase susceptibility to desiccation and erosion (Brennessel, 2006; Scholz, 2007). However, optimal natural nesting habitat is often limited in many parts of their range, and “preferred” nesting sites shift to altered habitats such as roadsides, residential yards, and agricultural fields (Butler et al., 2018; Feinberg & Burke, 2003). Females also exhibit strong nest philopatry, returning to the same nesting site each year (Brennessel, 2006; Butler et al., 2018).

Females move to, aggregate in, and likely emerge from nearshore areas adjacent to nest sites (Butler et al., 2018; Feinberg, 2004). Nesting is a critical part of nesting species' phenology, so it would be expected that distribution and abundance patterns may be linked to areas of higher nesting preference. Identifying and modeling environmental factors that influence spatiotemporal abundance patterns are key for more informative and useful estimates of population size, yet these factors are largely absent from DBT population studies. Population estimates of DBT are typically generated using capture–recapture methods (Avissar, 2006; Baxter et al., 2016; Butler, 2002; King & Ludlam, 2014). These methods generate valuable information on abundance, survivorship, and demography, but require substantial effort that often restricts sampling to only a few areas within the population region (Hart & McIvor, 2008; King & Ludlam, 2014). Moreover, sampling periods are often short in duration, lasting only a few weeks to a few months within the active season (Baxter et al., 2016; Simoes & Chambers, 1999). Abundance can then only be inferred within that spatial and temporal extent, which provides little information on abundance patterns within the larger population. Another monitoring approach, adapted from avian point count methods (Ralph et al., 1995), resolves the spatial and temporal limitations of capture–recapture through rapid, non‐invasive shore‐based visual headcount surveys. First, described by Harden et al. (2009), visual surveys were formally evaluated as a population assessment method by Levasseur et al. (2019) and produced estimates of occupancy and local abundance across a large spatial scale and over a long sampling period (e.g., majority of active season). Moreover, that study produced quantitative evidence of spatiotemporal variation in abundance, and although coarse in spatial and temporal resolution, interesting patterns still emerged, setting the stage for finer‐scale investigations.

Successful conservation and management interventions require understanding where, when, and how many individuals occur on the landscape. For example, knowledge of the locations and timings of DBT seasonal movements would allow for temporary use restrictions (e.g., crab trapping, shellfishing, and boating) and protection of important habitats, including saltmarsh and upland nesting areas. However, these high‐use areas can be easily missed if assessments occur outside of these areas (i.e., spatial) or take place before or after aggregation and staging periods (e.g., temporal). We suspect movement to, and selection of, nesting areas extends beyond just terrestrial habitats where eggs are laid. We hypothesize proximity to saltmarsh habitat will have a strong, positive association and proximity to developed habitats will have a strong negative association on DBT nest site selection.

We investigate proximity to suitable nesting habitat as a driver of spatiotemporal variation in local relative DBT abundance in Wellfleet Bay, Massachusetts, through two primary objectives. First, we investigate how habitat influences DBT nest site selection. We use a scale selection resource selection function (RSF) to identify the scale at which terrapins select for (or avoid) areas to nest, then use the top‐ranked scale of selection in a second covariate selection RSF to predict areas of suitable nesting habitat and create an index of nest suitability (NSI). Second, we test the influence of the hypothesized relationship of NSI on expected relative DBT abundance using a generalized linear mixed model (GLMM).

## 2 MATERIALS AND METHODS

### 2.1 Study area

**FIGURE 1:**
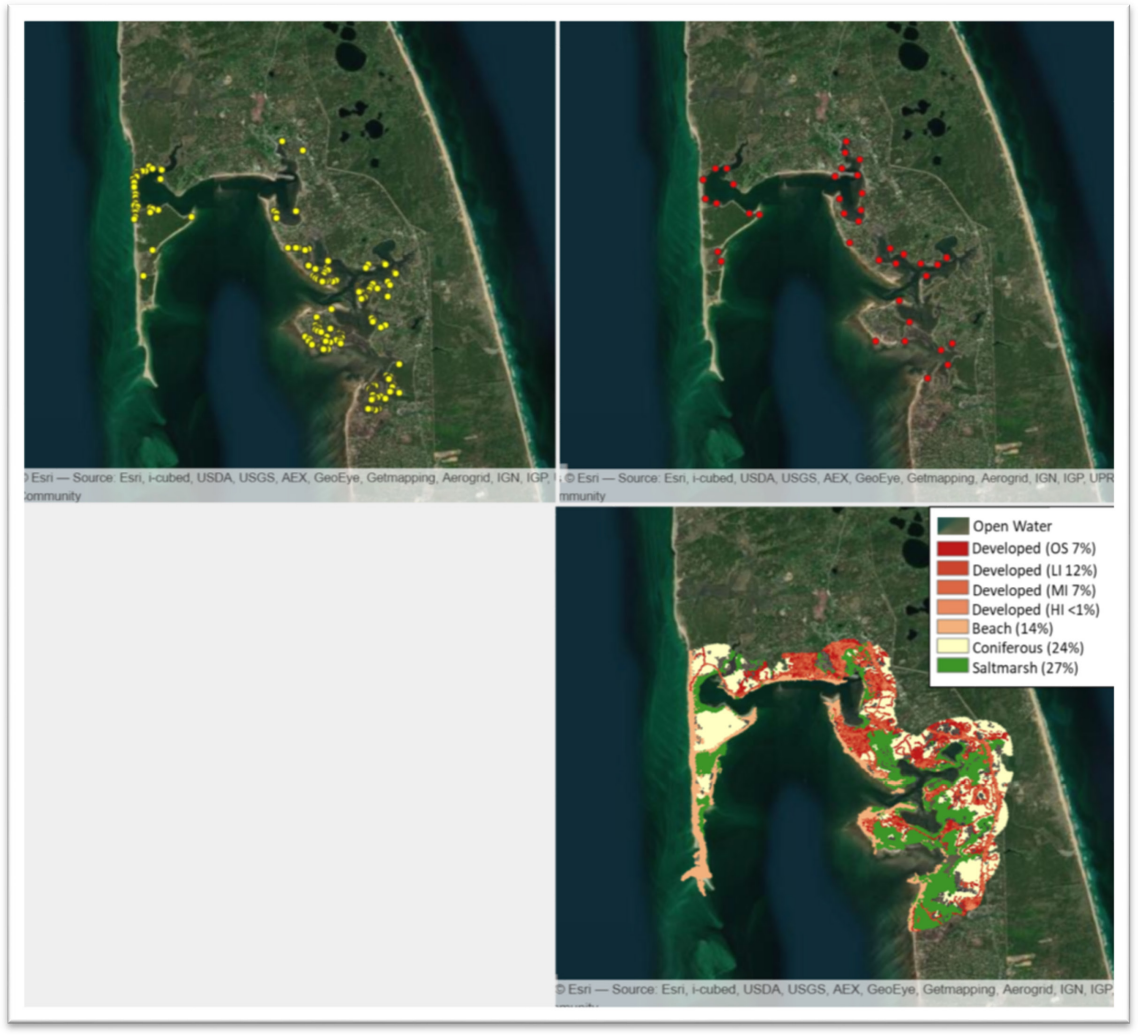
The Wellfleet Bay study area, including nest locations (*top left*; yellow circles), visual survey locations (*top right*; red circles), and NLCD landcover data (*bottom right*).

The study is focused on the town of Wellfleet (Cape Cod Bay, Massachusetts, USA) which hosts the northernmost concentration of the DBT across their range. Wellfleet Bay is a sheltered embayment with approximately 50 km of shoreline comprised of extensive saltmarsh habitat (Figure 1). We are specifically interested in the role of habitat in predicting nest site locations and the spatial distribution of individuals. We first identified our potential nesting landscape as all areas within 700 m from the shoreline. We used 700 m, which was twice the distance of the farthest nest location from the shoreline to ensure all possible nesting habitat was included. We then extracted habitat data for the area from the 2016 National Land Cover Database (NLCD, Multi‐Resolution Land Characteristics Consortium, 2021a, 2021b) using the Level 1 classification: *Open Water, Developed* (Open Space, Low Intensity, Medium Intensity, High Intensity), *Barren* (Sand), *Forest* (Deciduous, Evergreen, Mixed)*, Shrubland* (Shrub/Scrub), *Herbaceous* (Grassland/Herbaceous, Cultivated Crops), and *Wetlands* (Woody Wetlands, Emergent Herbaceous Wetlands) (Multi‐Resolution Land Characteristics Consortium, 2021b). Deciduous and Mixed Forest, Herbaceous and Woody Wetlands categories were each comprised of ≤3% of total landcover and were not included, and therefore we considered only *Open Water*, *Developed* (all four classes), *Emergent Herbaceous Wetland* (hereafter Saltmarsh), *Barren* (hereafter Beach), and *Evergreen Forest* (hereafter Coniferous) land use types in the analyses described below (Figure 1).

### 2.2 Nest site selection using resource selection functions

Diamondback terrapin nesting data were obtained from The Massachusetts Audubon Society, which has been collating records of nest locations collected by staff and volunteers since 2005 (Mass Audubon, 2021). Here, we use the nest location data from 2005 to 2018 which includes 952 usable nest locations. To avoid pseudo‐replication (i.e., same location being used in multiple years and spatially clustered nests), we divided the study area into 5 x 5 m pixels and generated binary incidences that indicated whether at least one DBT nest had been observed in a pixel over a 13‐year period. This reduced the number of nest locations from 952 to 560 pixels in which a nest was observed.

Resource selection functions are binomial generalized linear models that use the designation of used (y=1) versus available (y=0) as a response variable and habitat variables as predictors to estimate selection coefficients. In this analysis, pixels in which at least one nest was located were the used locations (the presences). Because search effort associated with the nest records has never been recorded, true absences are not available. As is traditional in RSF analyses, we generated “background zeros” (also known as pseudo‐absences) where a point was randomly sampled from any 5 x 5 m pixel that did not contain a true nest. The sampled points represent theoretically available locations where a terrapin *could have* chosen to nest within the study area. Following Renner et al. (2015), we performed a simulation to identify the appropriate number of available points, where the full covariate model was fitted with an increasing number of background zeros. Each fitted model was replicated 30 times to test the stability, or magnitude of change between coefficient estimates after each replication. Estimates are considered stable when there is little to no change in coefficient values between replicates. We found coefficient estimates stabilized at 75,000 available points (Appendix A: Figure S1), and hence our analysis consists of 560 used and 75,000 available points.

As predictors in the RSF, we summarized our landscape covariates of interest at the same resolution as the used and available locations (i.e., the same 5 x 5 m grid). We calculated the proportion of each pixel made up of: open water, developed, saltmarsh, beach, and coniferous forest landcover types. Specifically, we expect that DBT will select nest areas close to saltmarsh habitats as saltmarshes are the preferred habitat in northern latitudes, providing food, protection, and freshwater inputs (Brennessel, 2006). Moreover, staging and emerging females may favor the cover provided by saltmarsh vegetation (e.g., *Spartina alterniflora and S. patens*). We expect DBT will select beach habitats, which provide open areas and loose sandy soils preferred for egg deposition (Butler et al., 2018). We expect avoidance of developed areas, which are prone to more human activity and increased disturbance while attempting to nest (Brennessel, 2006; Isdell et al., 2015). We expect coniferous habitats to be predominately closed canopy and thus avoided by DBT (Palmer & Cordes, 1988). Lastly, the amount of open water present in a given area is an indicator of exposure, where areas with greater proportions of open water (and thus, smaller proportions of saltmarsh) are more exposed than areas with smaller proportions of open water (Levasseur et al., 2019). Therefore, we expect DBT to avoid nest sites proximal to areas of open water.

In addition to the habitat variables, we were also interested in the spatial scale at which terrapins are selecting areas of suitable nesting sites, and as such, we first conducted a multiscale RSF for each landcover type following the methods of Bauder et al. (2018). This approach allowed us to identify the most appropriate scale for each covariate before modeling the combined effects of all habitat covariates. The proportion of each landcover covariate was calculated using a buffer of increasing radius of 25 m, from 100 to 700 m (e.g., 100 m, 125 m, 150 m … 700 m, the max extent of the study area), around each used and available nest location. We then fit 26 binomial GLMs for each landcover covariate, i.e., a model at each scale and a null model, and compared these models using Akaike's information criterion (AIC, Burnham & Anderson, 2002). To then test hypotheses about factors that influence nest site selection, we used the AIC top‐ranked scale for each landcover covariate to generate a candidate model set that comprised all combinations of the five‐scale‐specific landcover covariates, resulting in a total of 25 candidate models for the resource selection analysis (Table 1).

**TABLE 1:** Model selection table for the 25 candidate RSF models ranked according to their AIC scores.

Selection	*K*	AICc	ΔAICc	ωAIC	ΣωAIC
p (~developed + saltmarsh + open water)	4	5599.58	0.00	0.83	0.83
p (~developed + saltmarsh + beach + coniferous + open water)	6	5602.92	3.34	0.16	0.99
p (~saltmarsh + beach + open water)	4	5608.99	9.41	0.01	1.00
p (~saltmarsh + open water)	3	5612.62	13.04	0.00	1.00
p (~developed + saltmarsh + beach + coniferous)	5	5742.48	142.90	0.00	1.00
p (~developed + saltmarsh + beach)	4	5746.92	147.34	0.00	1.00
p (~developed + saltmarsh)	3	5760.01	160.44	0.00	1.00
p (~developed + saltmarsh + coniferous)	4	5761.27	161.69	0.00	1.00
p (~saltmarsh + coniferous)	3	5765.10	165.52	0.00	1.00
p (~saltmarsh + beach + coniferous)	4	5765.56	165.98	0.00	1.00
p (~saltmarsh + beach)	3	5765.90	166.32	0.00	1.00
p (~saltmarsh)	2	5767.17	167.60	0.00	1.00
p (~developed + beach + coniferous)	4	5790.47	190.90	0.00	1.00
p (~developed + coniferous)	3	6120.11	520.53	0.00	1.00
p (~open water + coniferous	3	6198.44	598.86	0.00	1.00
p (~developed + beach + open water)	4	6231.72	632.14	0.00	1.00
p (~developed + open water	3	6259.95	660.37	0.00	1.00
p (~beach + coniferous)	3	6298.52	698.94	0.00	1.00
p (~developed + beach)	3	6307.29	707.71	0.00	1.00
p (~coniferous)	2	6354.21	754.63	0.00	1.00
p (~developed)	2	6398.56	798.98	0.00	1.00
p (~beach + open water)	3	6522.55	922.97	0.00	1.00
p (~open water)	2	6526.23	926.65	0.00	1.00
p (~beach)	2	6610.67	1011.09	0.00	1.00
p (~1)	1	6611.15	1011.58	0.00	1.00

*Note*: The selection (p) model formulations are provided, as is the number of parameters in the model (*K*), the AIC score, the difference in AIC relative to the top model (∆AIC), the AIC weight (ωAIC) which is a measure of relative model support, and the cumulative AIC weights (ΣωAIC).

All analyses were conducted in R (R Core Team, 2022). We used the package *stats* included in R (R Core Team, 2022) for calculating scale‐specific covariates and package *AICcmodavg* (Mazerolle, 2020) for AIC‐based model selection. Once the top RSF model had been identified, we generated a spatial prediction from the model using spatially explicit covariate values to produce a nest suitability index (NSI). This surface is a model prediction of the relative likelihood that a location is selected as a nest site.

### 2.3 Spatiotemporal variation in relative abundance

We used count data from visual headcount surveys conducted at 38 sites around Wellfleet Bay in the town of Wellfleet, MA, from May through October 2019, using the standardized protocol developed by Levasseur et al. (2019); (Figure 1). The surveys occurred at high tide and involved scanning the water with binoculars from shoreline left to shoreline right, recording the number of DBT heads observed within a 100 m radius of the survey point. Each survey consisted of five independent scans (e.g., counts) with a 1‐min break between each scan. Surveys were conducted at each site every 7–10 days for a total of 652 unique surveys.

We were interested in explaining spatiotemporal variation in (relative) DBT abundance and therefore, as the response variable, we used the maximum number of DBT observed in any of the scans in each survey. We analyzed these data using Poisson generalized linear mixed models (GLMMs), i.e., Poisson GLMs with an additional random effect term that accounts for the fact that each site was surveyed several times throughout the year (range: 14–20 visits). Preliminary analysis identified some overdispersion in the count data, likely due to excess zeros (zero inflation), and therefore, we used a zero‐inflated version of the Poisson generalized linear mixed model (GLMM).

We were specifically interested in testing the hypothesis that spatiotemporal variation in relative abundance was associated with the nest suitability index (*NSI*) produced from the resource selection modeling. The prediction here is that onshore habitat influences offshore abundance and therefore NSI is a proxy for suitable offshore habitat. To account for the seasonal variation, we included both day of season (Day) and its square (Day^2^) as temporal covariates. We generated a candidate model set of all combinations of the three predictor covariates including interactive effects of day (and its square) and NSI (Table 2). The rationale for the interactions is to allow not only for temporal changes in site‐specific abundance but also to allow for the effects of NSI to vary over time.

**TABLE 2:** Model selection table for the eight candidate zero‐inflated Poisson GLMMs ranked according to their AIC scores.

Relative abundance (expected mean count)	Zero inflation	*K*	AIC	ΔAIC	ωAIC	ΣωAIC
λ (~nsi*(day +day^2^) + (1| site))	p(~day +day^2^)	10	2806.98	0.00	0.91	0.91
λ (~day + day^2^ + (1| site))	p(~day +day^2^)	7	2813.28	6.29	0.04	0.95
λ (~nsi + (day +day^2^) + (1| site))	p(~day +day^2^)	8	2815.19	8.20	0.02	0.97
λ (~nsi*day + (1| site))	p(~day +day^2^)	8	2815.22	8.23	0.01	0.98
λ (~day + (1| site))	p(~day +day^2^)	6	2815.41	8.43	0.01	0.99
λ (~nsi + day + (1| site))	p(~day +day^2^)	7	2817.32	10.34	0.01	1.00
λ (~nsi + (1| site))	p(~day +day^2^)	6	3149.29	342.30	0.00	1.00
λ (~1+ (1| site))	p(~1)	3	3190.26	383.27	0.00	1.00

*Note*: The relative abundance (λ) including a random effect of site (1|site) and zero inflation (p) model formulations are provided, as is the number of parameters in the model (*K*), the AIC score, the difference in AIC relative to the top model (∆AIC), the AIC weight (ωAIC) which is a measure of relative model support, and the cumulative AIC weights (ΣωAIC).

The use of zero‐inflated GLMs allows the probability of being an excess zero to also be modeled using predictor covariates. We suspect the major source of the observed zero inflation is the movement reflecting seasonal ecology of the species. As such, we included day of season (Day) and its square (Day^2^) as predictors of zero inflation in all candidate models, noting also that site is included as a random effect (Table 2). As with the nest site selection, little is known about the spatial scale at which nearshore aggregating is selected and therefore we again opted for a scale selection approach to identify the representative scale. In this case, where NSI was the only covariate of interest, we identified the full spatiotemporal model as one containing a time effect (day of season) and its square to allow for flexibility to capture simple non‐linear temporal patterns using first‐order polynomial terms, and a spatially explicit effect, the NSI. We then compared models with NSI calculated at increasing buffers (scales) to determine the scale that maximally explained the variation in the data. As before, buffers ranged from 100 to 700 m at increasing intervals of 25 m (see above). Once we identified the AIC best scale for NSI, we proceeded to compare all eight models nested within the full model (Table 2). All analyses were conducted in R (R Core Team, 2022) using the package *glmmTMB* (Brooks et al., 2017) for model fitting, and package *AICcmodavg* (Mazerolle, 2020) for AIC‐based model selection.

## 3 RESULTS

### 3.1 Nest site selection using resource selection functions

**FIGURE 2:**
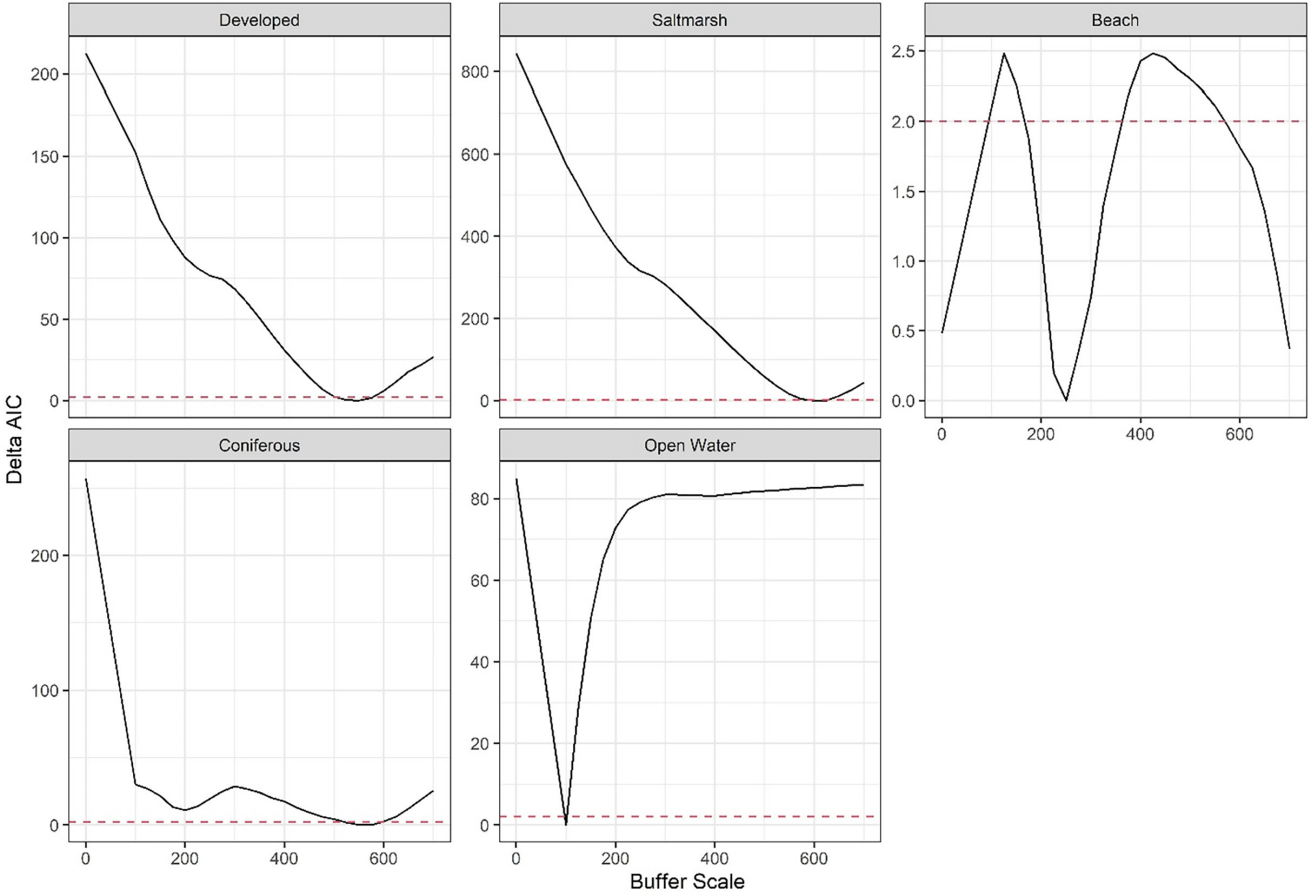
Change in ΔAIC across buffer scales for each landcover covariate. Red dashed line indicates Δ2 AIC units. Top‐ranked buffer scales have a ΔAIC score of 0.

For the multiscale RSF, based on model selection using AIC, the best‐supported model had DBT selection for developed, coniferous, and saltmarsh land use classes to be at a scale of 550, 575, and 600 m, respectively, and a scale of 250 m for beach and 100 m for open water land use classes (Figure 2).

**FIGURE 3:**
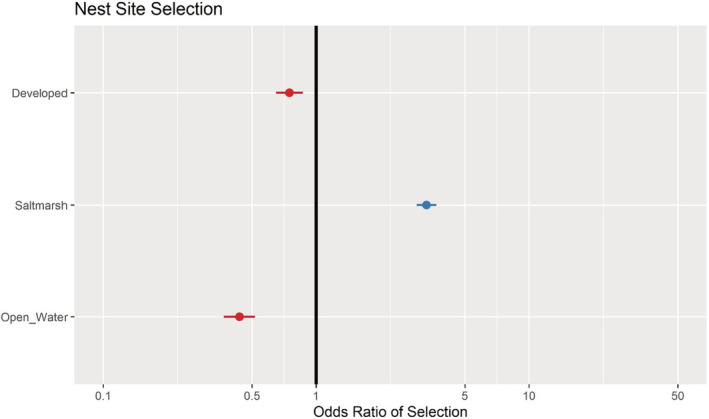
Odds ratio plot for nest site selection for each habitat covariate. Values greater than 1 (blue circles) represent selection for that habitat type and values less than 1 (red circles) represent avoidance of that habitat type. Horizontal bars extending out from each point represent 95% confidence interval.

**FIGURE 4:**
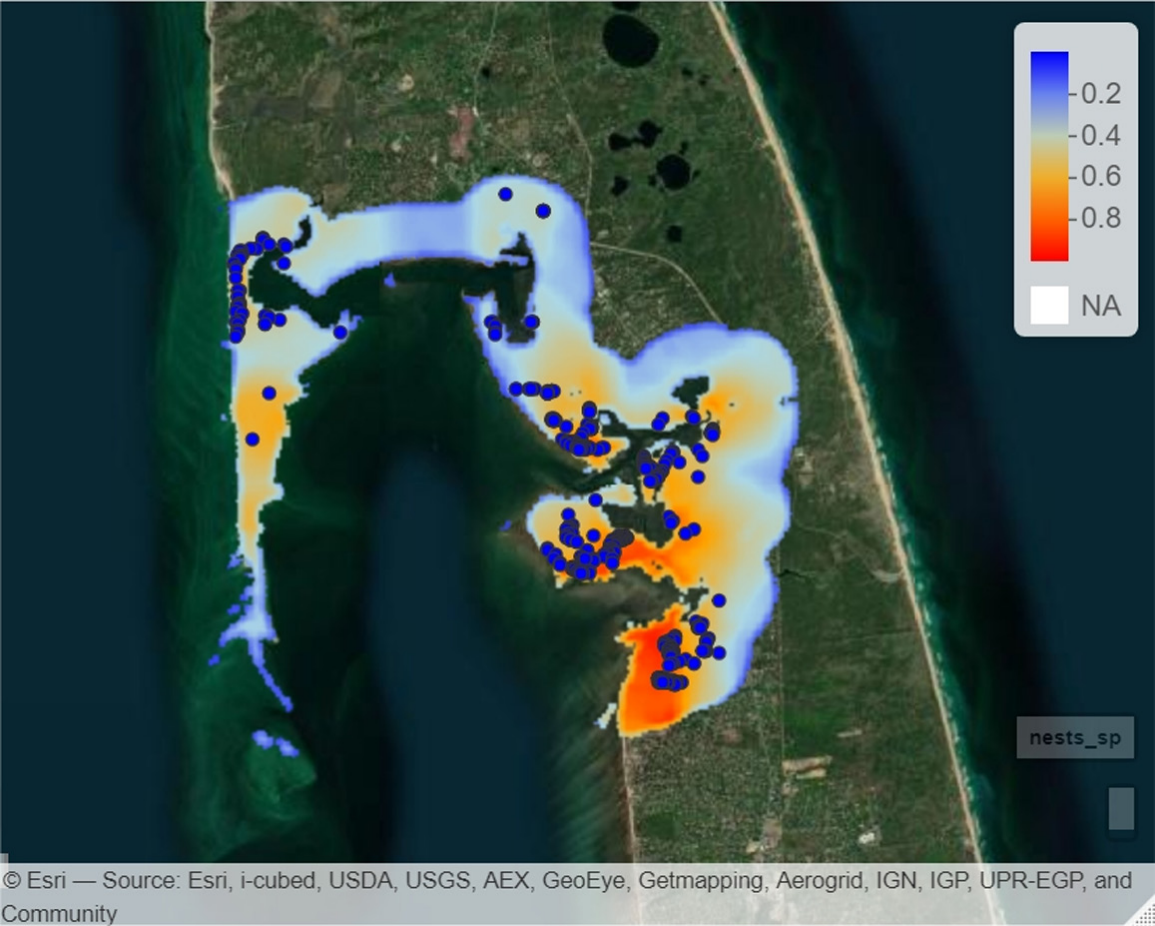
Predictive likelihood surface map of suitable nesting habitat around Wellfleet Bay within the town of Wellfleet with a 0–1 probability scale. Blue circles denote nest locations.

Using the top‐ranked scale of selection for each landcover type and subsequent model selection using AIC, the best‐supported model found nest site selection to be influenced by the proportion of developed, saltmarsh, and open water landcover types (Table 1). Here, we report the odds ratio of selection, which represents the constant effect of each landcover type on the likelihood of a nest being present, with θ>1 indicating a positive effect (selection for) and θ<1 indicating a negative effect (selection against). Specifically, there was selection for saltmarsh of 3.30 (CI: 2.96–3.66), whereas there was avoidance of developed (0.75; CI: 0.65–0.87) and open water (0.44; CI: 0.37–0.52; Figure 3). Using the results top model, a predictive likelihood surface map was created of the relative probability of suitable nesting habitat within areas of interest (Figure 4).

### 3.2 Spatiotemporal variation in relative abundance

**FIGURE 5:**
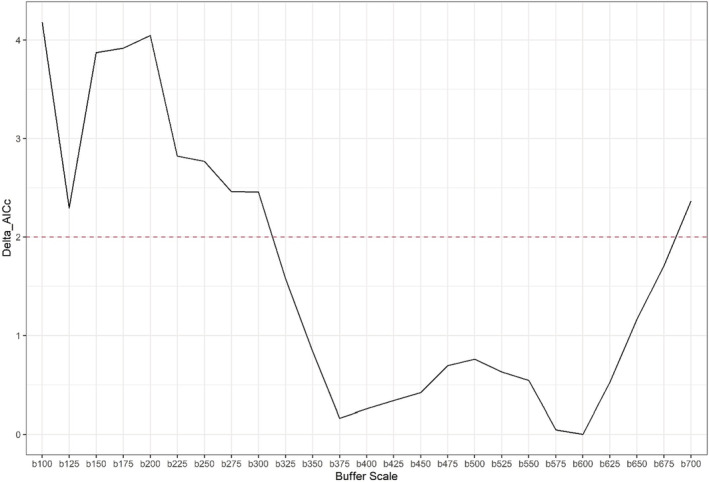
Change in ΔAIC across buffer scales for NSI. Dashed line indicates Δ2 AIC units. Top‐ranked buffer scales have a ΔAIC score of 0.

For the multiscale zero‐inflated GLMM, based on model selection using AIC, the best‐supported model found an NSI buffer scale of 600 m to maximally explain variation in the data (Figure 5).

**FIGURE 6:**
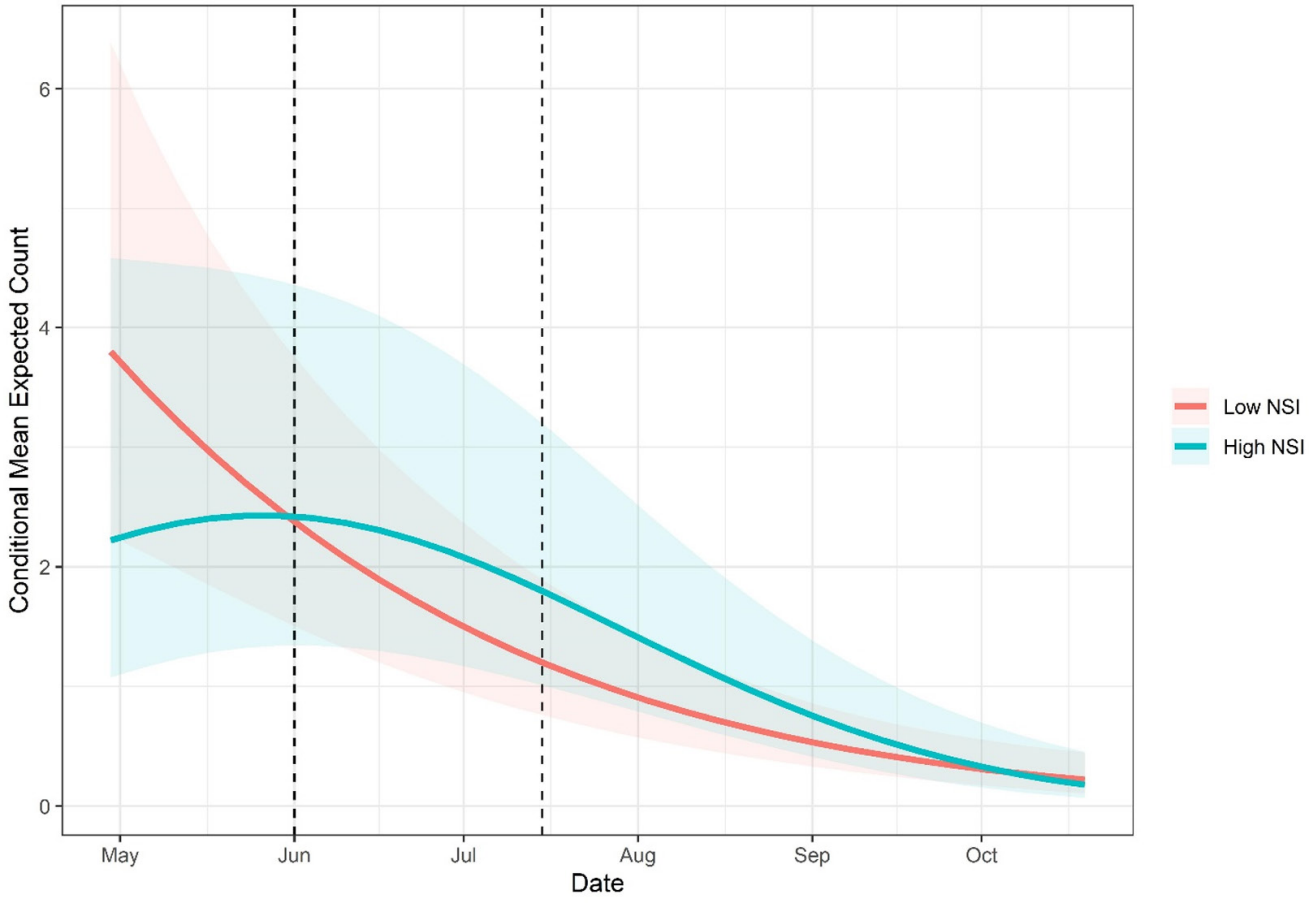
Mean expected counts through the season at survey sites within low NSI (red line) and high NSI (blue line) Areas. Shaded regions within each plot represent 95% confidence intervals and black vertical dashed lines indicate approximate peak nesting season (Jun 1 to Jul 15).

**FIGURE 7:**
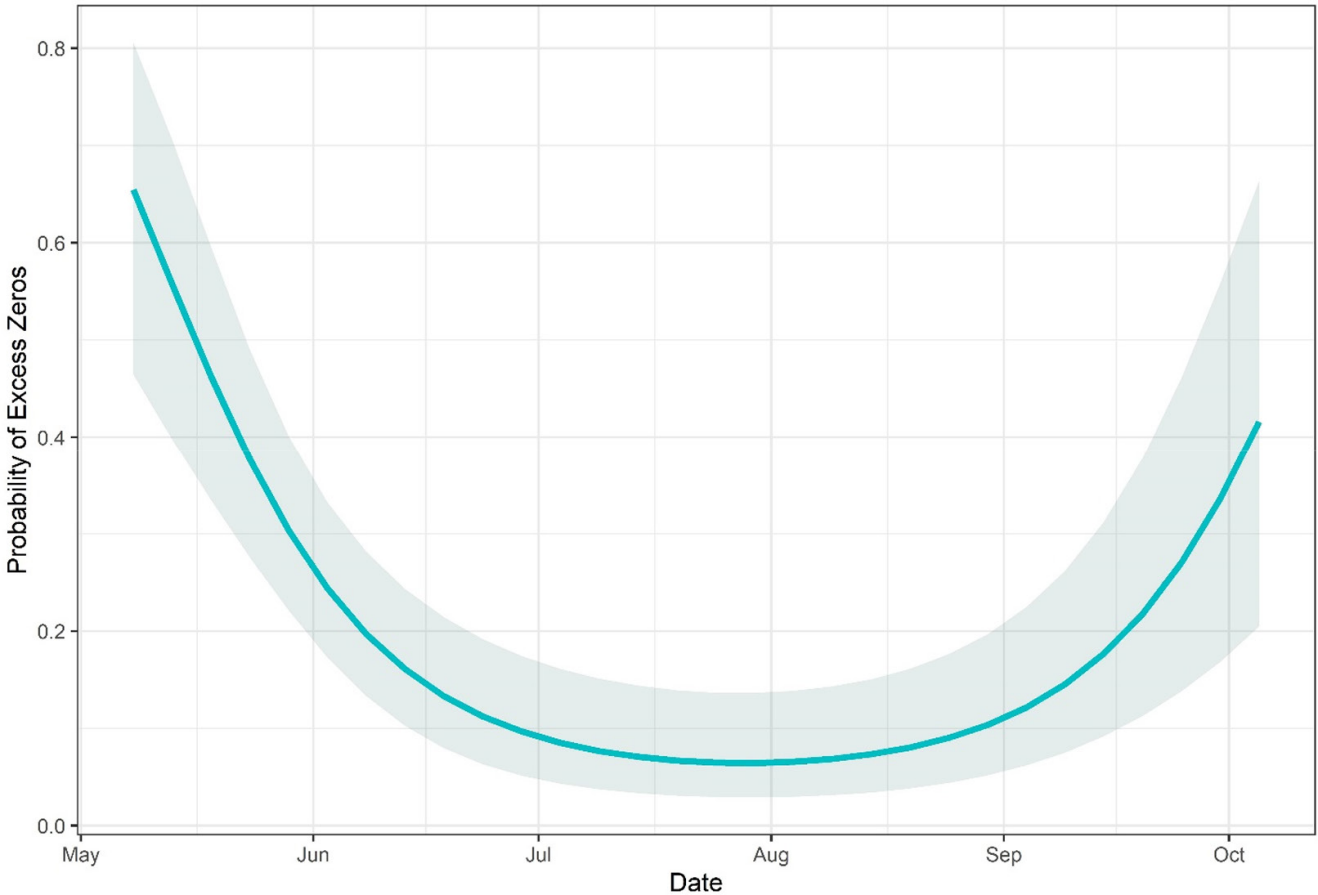
Probability of excess zeros in the count data. Shaded region represents 95% confidence intervals.

Using the top‐ranked NSI buffer scale and based on model selection using AIC, the best‐supported model found expected relative abundance to be influenced by the interaction between NSI and day of season $\lambda \;\left(\mathrm{NSI}*\mathrm{Day}+\text{Nest}*{\mathrm{Day}}^{2}\right);\;$ (Table 2). Specifically, NSI (0.16, SE: 0.18, *p* = .37) and the interaction of NSI and Day (0.02, SE: 0.04, *p* = .68) both positively influenced expected relative abundance but were not significant effects in the model. In contrast, Day (−0.69, SE: 0.05, *p* = <.00) and Day^2^ (−0.10, SE: 0.04, *p* = .02) had the largest significant negative influence on expected relative abundance, resulting in a humped‐shaped response (Figure 6). This was followed by the interaction of NSI and Day^2^ (−0.11, SE: 0.04, *p* = .01). For example, at the beginning of the sampling season on May 8th, expected relative abundance was slightly higher at sites adjacent to low predicted nest suitability (NSI) areas (3.80, CI: 2.17–6.67) than at sites adjacent to areas of high NSI (2.15, CI: 1.10–4.21, Figure 6). However, once into the nesting season around June 18th, the pattern of expected relative abundance reverses, with an increase at high NSI sites (2.20, CI: 1.20–3.93) and a decrease at low NSI sites (1.84, CI: 1.10–3.10, Figure 6). Lastly, there is gradual decrease in expected relative abundance throughout the season within both high and low NSI sites although the decline is faster in low‐NSI areas (Figure 6). The zero‐inflated model, which used seasonality as a predictor, found Day to have a negative but insignificant effect on the probability of excess zeros (−0.16, SE: 0.17, *p* = .36) and Day^2^ to have a positive and significant effect (0.93, SE: 0.21, *p* = <0.01), resulting in a hull‐shaped response (Figure 7). Specifically, the probability of excess zeros was found to be highest at the beginning and end of the sampling season and lowest during the middle of the sampling season (Figure 7). For example, the probability of surveys conducted on May 8th and October 5th containing excess zeros in the count data are 0.65 (CI: 0.46–0.80) and 0.42 (CI: 0.20–0.66), respectively. In contrast, the probability of excess zeros within surveys conducted on July 29th is 0.06 (CI: 0.03–0.14, Figure 7).

## 4 DISCUSSION

In this study, we present the first evaluation of suitable nesting habitat and its potential effects on spatiotemporal variation in relative abundance within the northernmost populations of a threatened saltmarsh specialist. Using nest location data and NLCD landcover data, we found the scale of selection for each landcover type to be at two very different spatial scales. Saltmarsh (600 m), coniferous (575 m), and developed (550 m) had selection scales approximately two times larger than beach (250 m) and five to six times larger than open water (100 m) landcover types (Figure 2). Nest habitat selection by females was positively associated with the presence of adjacent saltmarsh and negatively associated with adjacent developed and open water landcover types (Figure 3). Moreover, we found that offshore relative abundance was positively associated with our estimated metric of nest suitability (NSI) during the nesting season.

For saltmarsh and developed landcover types, the spatial scale and selection are intuitive. Proximity to saltmarsh (or proportion surrounding a nest) should not be important at small scales, since nests are deposited upland of marsh habitat above the mean high water line (Feinberg & Burke, 2003; Roosenburg et al., 2014). However, the presence of saltmarsh is important at larger spatial scales, as it is important foraging habitat and sheltered from wave action (Brennessel, 2006; Tucker et al., 2018). Moreover, saltmarsh vegetation may serve as important cover for staging females, although this has yet to be formally evaluated. Similarly, at local scales, developed areas typically lack suitable nesting habitat (although DBT in Wellfleet are known to nest in altered landscapes such as lawns, dirt roads, and driveways) and are subject to high levels of anthropogenic disturbance during nesting attempts (Butler et al., 2018; Robert Prescott, R. P. pers. obs; Wnek et al., 2013). Moreover, highly developed areas are more likely to lack suitable offshore habitat (e.g., saltmarsh) and would therefore be avoided by DBT at larger spatial scales (Bilkovic et al., 2012). Indeed, we found support for avoidance of developed areas within Wellfleet Bay (Figure 1). Avoidance of open water landcover at local scales is expected given that it is a measure of exposure (i.e., linear shoreline open to the larger bay) that is inversely proportional to saltmarsh landcover and subject to increased offshore turbulence and disturbance (Brennessel, 2006; Levasseur et al., 2019). We expected selection for beach landcover, given established nest site substrate preferences and nest philopatry (Goodwin, 1994; Roosenburg & Place, 1995), however, beach did not appear in our top model. This may be a result of beaches comprising only 14% of the total NLCD landcover within the study area and including barrier beaches and other stretches that are low lying and subject to overwash (e.g., flooded with saltwater from severe weather), thus not suitable for nesting (Figure 1, Prescott, pers. com). Likewise, coniferous landcover did not appear in our top model, suggesting coniferous forests are not a strong indicator of nest site selection for the Wellfleet Bay population.

Our results demonstrate that RSFs are a useful tool in identifying patterns of resource use, and in our case, identifying areas of suitable nesting habitat by making predictions about where DBT are likely to nest within Wellfleet Bay. The data we used are, however, not systematic surveys and thus have some biases. We have attempted to address these biases using recommended RSF modeling practices and while we believe that our findings are intuitive and in line with a range of predictions, we recommend using our results as the basis for further hypothesis testing using standardized data and a design specifically suited to testing hypotheses about nest site selection. Moreover, during this work, we found generating a suite of landscape covariates for dynamic coastal zones to be challenging, and this too is an area of future research that will likely benefit the investigation of onshore resource selection by nesting aquatic/marine species.

The objective for the abundance analyses using GLMMs was to investigate the influence of suitable nest habitat (NSI), as predicted by the RSF analysis, on variation in relative abundance. For the count model, we found that the interaction of day of season and NSI best explained variation in relative abundance (Table 2). Consistent with the results of Levasseur et al. (2019), we find a negative temporal trend in expected counts as the season progresses, however, with the addition of a spatial habitat covariate, we see a difference in the shape and magnitude of that trend. With the interaction effect of day of season, higher expected relative abundance shifted from sites with a low NSI to sites with a high NSI during the nesting season (Figure 6). Moreover, relative abundance remained higher and declined slower at sites with high NSI (Figure 6). As expected, the zero‐inflated model found temporal variation in the probability of excess zeros within the count data, with the highest probability during the beginning (May) and end (October) of the sampling season (Figure 7). This pattern is in line with DBT ecology at their northern latitudes, where late April and October correspond to the start and near end of the active season (Castro‐Santos et al., 2019). The zero‐inflated model can be thought of as a proxy for detection, where in the early spring and late fall, temperatures are colder and DBT are less active on average. This can result in DBT being present at a site, but going undetected due to their inactivity and therefore contributing more excess zeros in the data than would be expected.

Our expected counts are small (range: 0.2–3.8 individuals) and that is a result of the limitations of the model used in this study. GLMMs do not contain explicit models for detection and, as such, are only measures of the expected number of individuals to be counted at a site (e.g., relative abundance) and are not measures of true abundance. However, because we were interested in the relationship between a spatial nest habitat covariate and expected counts, this was an appropriate modeling framework. Although significant, the effects of NSI and day of season are small and could be a result of the count data also including males and subadults in addition to females, weakening the effect. It is also possible that variations in expected counts are more related to tidal variations or suitable aquatic habitat or perhaps a combination of habitat variables.

Although careful in our inference on DBT nest site selection, we argue the patterns of selection are informative, particularly in regards to the importance of nearby saltmarsh habitats when prioritizing areas for nest site protection, restoration, or creation. Moreover, our results support proximity to nesting habitat has some influence on spatiotemporal variation in relative abundance and should be further investigated in true abundance models. Including carefully chosen environmental covariates provides the opportunity to extend beyond local estimates of abundance at each site and begin to relate spatial variation in those abundance estimates to specific ecological characteristics at a finer scale. From a conservation and management perspective, local abundance estimates from visual headcount data tracked through space and time allow for the monitoring of terrapin populations and detection of decline or recovery at much larger scales. Likewise, linking abundance and distribution patterns to specific ecological drivers such as aggregation sites, nesting habitat, and prey availability can provide the spatiotemporal information needed for appropriate and effective management decisions.

## AUTHOR CONTRIBUTIONS

**Patricia Levasseur:** Conceptualization (lead); data curation (lead); formal analysis (lead); funding acquisition (lead); investigation (lead); methodology (lead); project administration (lead); validation (lead); visualization (lead); writing – original draft (lead); writing – review and editing (lead). **Robert Prescott:** Data curation (supporting); funding acquisition (supporting); investigation (supporting); validation (supporting); writing – review and editing (supporting). **Mark Faherty:** Data curation (supporting); funding acquisition (supporting); investigation (supporting); validation (supporting); writing – review and editing (supporting). **Chris Sutherland:** Conceptualization (supporting); formal analysis (equal); investigation (supporting); methodology (equal); supervision (lead); validation (supporting); visualization (supporting); writing – original draft (supporting); writing – review and editing (supporting).

## Supporting information


Figure S1

## Data Availability

The data that support the findings of this study are available on request from the corresponding author. The data are not publicly available due to privacy or ethical restrictions.
