# Supplementary material for: Factors affecting spatiotemporal patterns of nest site selection and abundance in diamondback terrapins
Source: Ecol Evol. 2023 Mar 15;13(3):e9866. doi: 10.1002/ece3.9866 (PMC10017312; doi:10.1002/ece3.9866)
Supplement: Supplementary file 1 — Figure S1 [file ECE3-13-e9866-s001.docx]

**Appendix A**


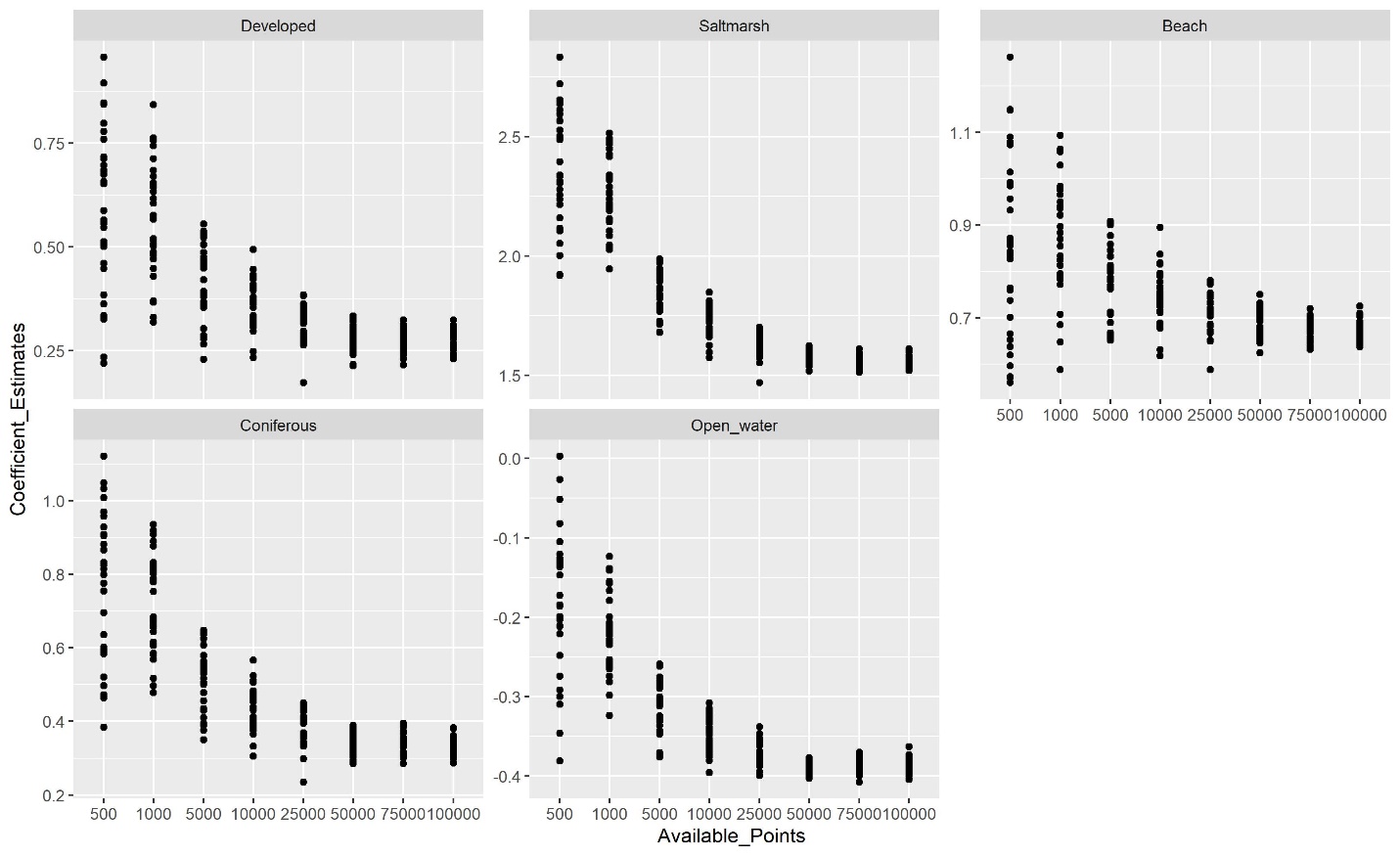


**Figure S1.** RSF (binomial GLM) simulation of the nests dataset to identify the appropriate number of available points. The full covariate model was fitted with an increasing number of randomly chosen available points from 500 to 100,000 and replicated 30 times to test the stability of coefficient estimates. Estimates stabilized at 75,000 randomly chosen available points.
